# An update on explaining the rural-urban gap in under-five mortality in India

**DOI:** 10.1186/s12889-022-14436-7

**Published:** 2022-11-16

**Authors:** Chandan Kumar, Piyasa, Nandita Saikia

**Affiliations:** 1 International Institute for Population Sciences, Mumbai, Maharashtra 400088 India; 2 Department of Public Health and Mortality Studies, International Institute for Population Sciences, Govandi Station Road, Deonar, Mumbai, Maharashtra 400088 India

**Keywords:** Economic differential, India, NFHS-5, Rural-urban gap, Under-five mortality

## Abstract

**Background:**

Rural Indians have higher mortality rates than urban Indians. However, the rural-urban gap in under-five mortality has changed is less researched. This paper aims to assess 1) whether the rural-urban gap in under-five mortality has reduced over time 2) Whether rural children are still experiencing a higher likelihood of death after eliminating the role of other socioeconomic factors 3) What factors are responsible for India’s rural-urban gap in under-five mortality.

**Methods:**

We used all rounds for National Family Health Survey data for understanding the trend of rural-urban gap in under-five mortality. Using NFHS-2019-21 data, we carried out a binary logistic regression analysis to examine the factors associated with under-five mortality. Fairlie’s decomposition technique was applied to understand the relative contribution of different covariates to the rural–urban gap in under-five mortality.

**Results:**

India has witnessed a more than 50% reduction in under-five mortality rate between 1992 and 93 and 2019–21. From 1992 to 93 to 2019–21, the annual decrease in rural and urban under-five mortality is 1.6% and 2.7%, respectively. Yet, rural population still contributes a higher proportion of the under-five deaths. The rural-urban gap in under-five mortality has reduced from 44 per thousand live births in 1992–1993 to 30 per thousand in 2004–2005 which further decreased to 14 per thousand in 2019–2021. There is no disadvantage for the rural children due to their place of residence if they belong to economically well-off household or their mothers are educated. It is wealth index rather than place of residence which determines the under-five mortality. Economic (50.82% contribution) and educational differential (28.57% contribution) are the main reasons for rural-urban under-five mortality gaps.

**Conclusion:**

The existing rural-urban gap in under-five mortality suggests that the social and health policies need to be need to reach rural children from poor families and uneducated mothers. This call for attention to ensure that the future programme must emphasize mothers from economically and educationally disadvantaged sections. While there should be more emphasis on equal access to health care facilities by the rural population, there should also be an effort to strengthen the rural economy and quality of education.

**Supplementary Information:**

The online version contains supplementary material available at 10.1186/s12889-022-14436-7.

## Background

Since 1990, child mortality has been reducing substantially. The worldwide under-five mortality rate has dropped by 60%, from 93 deaths (12.6 million) per 1000 live births in 1990 to 37 deaths (5.0 million) per 1000 live births in 2020 [1, 2]. Yet, the gaps in under-five mortality between countries are unacceptably wide. According to WHO (2020), children from low-income countries such as sub-Saharan Africa continued to have the highest mortality rates in the world at 74 (68 to 86) deaths per 1000 live births, which is 14 times higher than the risk for children in Europe and North America. Interestingly in some countries, the gap in under-five mortality within country is stark. One such country is India, where there is wide variation in under-five mortality by socioeconomic and regional characteristics. India was identified as a high disparity country on absolute and relative scales on under-five mortality [3]. According to nationally representative data NFHS-5 (2019–2021), there is a considerable variation in the under-five mortality rate in different states of India. The highest under-five mortality rate per thousand is observed in Uttar Pradesh (60); Chhattisgarh (50); Madhya Pradesh (49); Jharkhand (45); Odisha (41); Rajasthan (37) and whereas the low under-five mortality rate states are Tamil Nadu (22); Kerala (5). In India, the caste differential in under-five mortality is found to be minimal whereas, the under-five mortality among the poorest wealth quintile (59) is thrice higher than that of the richest wealth quintile (20).

United Nations Millennium Development Goals (2000) aimed to reduce under-five mortality by two-thirds (MDG 4) by 2015, but many poor countries could not achieve the target and were labelled as ‘off-track’, ‘insufficient progress’, or ‘no progress ’[4]. Poor countries faced many challenges to achieving this target, such as lack of health care infrastructure and health professionals, lack of resources and technology, vaccines, lower literacy rate, low household wealth, whereas rich countries increase household wealth and mother literacy, which help to reduce under-five mortality [5]. The call for SDGs with the motto ‘No one left behind,’ attempted to reduce inequalities across gender, region, class, and caste. The proposed SDG target for under-five mortality aims to reduce at least as low as 25 deaths per 1000 live births by 2030. Prior studies suggest that India had the largest number of under-five deaths of all countries in 2015, with substantial subnational disparities and the enormous absolute disparities [6]. While India achieved MDG on child mortality at the national level, many states,regions and some socioeconomic groups lagged behind to achieve it. A previous study found that while 9% of the districts had already reached the neonatal mortality rate (NMR) targeted in SDG3, nearly half (315 districts) were not likely to achieve the 2030 target even if they realized the neonatal mortality reductions achieved by their own states between NFHS 3 and 4 [7].

Interestingly, rural Indians experience always higher mortality than urban Indians. There are considerable rural-urban infant mortality differentials existed at the national and state levels irrespective of the level of the mortality. It was found that wide disparity in socioeconomic and community-level factors was the reason for rural-urban gap in mortality [8]. It should be noted that there are continuous efforts to reduce under-five mortality particularly in rural India through the intervention of different programs like the National Health Mission and other initiatives such as Janani Shishu Suraksha Karyakram (JSSK), Rashtriya Bal Swasthya Karyakram (RBSK), Mother and child health wings (MCH Wings), District hospital and knowledge center (DHKC), National Iron+ Initiative, National ambulance services, National Mobile Medical Units (NMMUs), Poshan Abhiyan etc. Yet, a quick glance at the rural-urban gap in under-five mortality shows that rural-urban gap still exists in high and low mortality states such as Chhattisgarh (27), Uttar Pradesh (13), Madhya Pradesh (14), Orrisa (11), Rajasthan (6), Jharkhand (28) and Tamil Nadu (9). It is imperative to investigate whether “place of residence” creates any mortality divide and if so, it is crucial find out the factors behind such gap to fulfil the SDG motto “No one left behind”.

The majority of under-five deaths in India are in rural India due to higher population share in urban area and, high under-five mortality rates in rural India. However, there is no recent study examining the recent dynamics in rural-urban gap in under-five mortality. This study aims to examine 1) whether the rural-urban gap in under-five mortality has reduced over time or not 2) Are rural children still experiencing higher likelihood of death after eliminating the role of other socioeconomic factors? 3) What are the factors responsible for rural–urban gap in under-five mortality in India.

## Methods

 To examine the trends in under-five mortality by place of residence, we used all round of National Family Health Survey (NFHS) data, whereas to investigate factors affecting the rural-urban gap in under-five mortality, we used data from 2019 to 21 (NFHS-5).

The National Family Health Survey (NFHS) provides the state as well as national estimates of fertility, infant and child mortality, the practices of family planning, maternal and child health, and nutrition of women and children. The NFHS 1992–93 (NFHS-1) comprises interviews with 88,562 households and 89,777 ever-married women aged 13–49. NFHS-2 (1998–99) covered 91,196 households and 89,199 ever-married women aged 15–49. The third round of NFHS 2005–06 (NFHS-3) interviewed 124,385 women aged 15–49 and 74,369 men aged 15–54. NFHS-4 (2015–16) interviewed with 572,000 households 699,686 women (aged 15–49) and 122,051 men (aged 15–54). The most recent round, the NFHS-5 survey for India was conducted in Phase-I from 17 June 2019 to 30 January 2020, covering 17 states and 5 uts & Phase II from 2 January 2020 to 30 April 2021, covering 11 states and 3 uts. They comprised of interviews from 636,699 households with a response rate of 98%., 724,115 women with a response rate of 97% and 101,839 men with a response rate of 92%. All the five rounds of NFHS collected detailed information on health, birth histories and related information on mothers and children. The birth history data allows for estimates of under-five mortality and examination of factors associated with under-five mortality in India, states and its districts.

The NFHS-5 sample is two stage stratified sampling technique. The district was grouped into rural and urban areas. For rural areas, substrata were created based on the estimated number of households in each village and the percentage of the population belonging to Scheduled castes (SC) and Scheduled tribes (ST). Before selection, psus were sorted to the literacy rate of women aged six or more years. Before selection, Primary sampling units (PSUs) were sorted in urban areas based on the SC/ST population percentage. The final sample PSUs were selected with Probability proportional to size (PPS) systematic sampling.

### Outcome variable

In the analysis, we used the information on births in five to 15 years preceding the survey date in NFHS-5. Thus, our analysis is based on a sample of babies born 60–180 months prior to NFHS-5 survey dates. NFHS asked question to the women who have ever given live birth “Have you ever given birth to a boy or girl who was born alive but later died?” If the answer is “died”, then asked “How old was when he/she died?” In this study, if the child died before age 59 months, it is considered under-five mortality. Our outcome variable “under-five Mortality” was assigned a value of 1 if the child died before age 59 months and 0 if the child was alive at least until age 59 months.

### Independent variable

The study used place of residence as the main independent variable. It is a binary variable taking values 0 for rural and 1 for urban.

### Control variables

Numerous studies have shown the role of several variables in explaining mortality [9–17]. The socioeconomic variables included in the models were mother’s education (no education, primary, secondary, higher), religion (Hindu; Muslim; Christian; and others), caste (Scheduled caste; Scheduled tribe; Other backward classes; others), wealth index (poorest; poorer; middle; richer; richest) and mass media exposure (Not exposed; partially exposed; exposed).

The demographic variables included in the models were the sex of the index child (male, female), birth interval (firstborn; second order birth and birth interval less than 24 months; second-order birth and birth interval equal to or more than 24 months; third higher-order birth and birth interval less than 24 months; third or higher order birth and birth interval equal to or more than 24 months); mother’s age at birth (less than 20 years, 20–29 years, more than 29 years) of the index child.

The region is an important determinant of mortality outcomes in India [7, 13, 18–20]. This variable is divided into six categories based on the NFHS-5 classification. These regions are south India (Andhra Pradesh, Karnataka, Telangana, Kerala, Lakshadweep, Tamil Nadu, Puducherry, Andaman and Nicobar island), North-East India (Sikkim, Arunachal Pradesh, Assam, Nagaland, Manipur, Mizoram, Tripura, Meghalaya) East India (West Bengal, Odisha, Jharkhand, Bihar) Central India (Madhya Pradesh, Uttar Pradesh, Chhattisgarh) North India (Jammu & Kashmir, Ladakh, Himachal Pradesh, Punjab, Haryana, Chandigarh, Rajasthan, Delhi, Uttarakhand) West India (Dadra & Nagar Haveli, Daman & Diu, Goa, Gujarat, Maharashtra).

### Statistical analysis

We carried out a binary logistic regression analysis to examine the factors associated with under-five mortality. Three different sets of models were carried out to examine the effects of ‘place of resident’ on the outcome. This was done to understand the pathway through which ‘place of resident’ affects the outcome of interest. Model 1 gives the effect of ‘place of resident’ on the outcome. Model 2 includes ‘place of resident’ and ‘demographic and socio-cultural’ variables. The wealth index is added in Model 3, along with the variables used in Model 2.

After that, we used Fairlie decomposition to decompose the rural-urban gap in under-five mortality by the exposure variables. The blinder-Oaxaca decomposition technique is commonly used approach to identify and quantify the factors associated with inter-group differences in the mean level of outcome. In this study, we have used this analysis to show how differences between the groups can explain differences in the under-five mortality between the rural and urban populations. This technique, however, is not appropriate if the outcome is binary (as in our case) in nature [21]. However, we used the extension of the Blinder–Oaxaca decomposition technique modified for binary outcomes to decompose the gap between the rural-urban in under-five mortality. Blinder–Oaxaca decomposition is a regression-based decomposition analysis.

According to Standard Blinder-Oaxaca decomposition, the rural-urban gap in the average value of the dependent variable, Z, (Under-five Mortality) can be expressed.

1 $${z}^{-R}-{z}^{-U}=\left[\left({k}^{-R}-{k}^{-U}\right){\hat{\beta}}^R\right]+\left[{k}^{-U}\left({\hat{\beta}}^R-{\hat{\beta}}^U\right)\right]$$

Where *k*^−*t*^ Is a row vector of average values of the independent covariates and ${\hat{\beta}}^t$ Is a vector of coefficient estimates for the type of residence t, an extension of this decomposition for a non-linear equation, Z= $\Big(k\hat{\beta \Big)}$, can be written the.

2 $${z}^{-R}-{z}^{-U}\left[{\sum}_{i=1}^{N^R}\frac{F\left({k}_i^R{\beta}^{-R}\right)}{N^R}-{\sum}_{i=1}^{N^R}\frac{F\left({k}_i^U{\beta}^{-R}\right)}{N^U}\right]+\left[{\sum}_{i=1}^{N^U}\frac{F\left({k}_i^U{\beta}^{-R}\right)}{N^U}-{\sum}_{i=1}^{N^U}\frac{F\left({k}_i^U{\beta}^{-U}\right)}{N^U}\right]$$

An equally valid expression for decomposition is,

3 $${z}^{-R}-{z}^{-U}=\left[{\sum}_{i=1}^{N^R}\frac{F\left({k}_i^R{\hat{\beta}}^U\right)}{N^R}-{\sum}_{i=1}^{N^U}\frac{F\left({k}_i^U{\hat{\beta}}^U\right)}{N^U}\right]+\left[{\sum}_{i=1}^{N^R}\frac{F\left({k}_i^R{\hat{\beta}}^R\right)}{N^R}-{\sum}_{i=1}^{N^R}\frac{F\left({k}_i^R{\hat{\beta}}^U\right)}{N^R}\right]$$

Here we define *Z*^*t*^ As the average probability of the binary outcome of the interest group j and F is the cumulative distribution function from the logistic distribution. Here ‘R’ stands for rural, ‘U’ stands for urban and ‘N’ stands for sample size. The first terms in eq. (2) and (3) provide an estimate of the contribution of rural-urban differences in the entire set of independent covariates to the rural-urban gap in under-five mortality. To find the total contribution, we need to calculate two sets of predicted probabilities by rural-urban and take the differences between the average values of the two.

However, identifying the contribution of group differences in specific covariates to the rural-urban gap is not straightforward. Usually, the sample sizes of the two groups are not the same, therefore one needs to follow these steps: -.

➣ First carry out regression for the combined data (rural and urban together) and calculate the predicted probabilities ${\hat{Y}}_i$, for each rural and urban observation in the sample.

➣ Since rural sample is bigger than urban sample, draw a random subsample of rural equal in size to the full urban Sample (*N*_*U*_)

➣ Each observation in the rural sample and full urban sample is then separately ranked by predicted probabilities and matched by their respective ranking. This procedure matches the rural under-five children who have characteristics placing them at the bottom (top) of their distribution with urban under-five children who have characteristics placing them at the bottom (top) of their distributions. Now assume that Nu = Nr and a natural one-to-one matching of urban and rural observations exists. Also assume that there are two independent variables to explain the rural-urban gap in under-five mortality (k_1_ and k_2_).

Now, according to Fairlie (2006), using coefficient estimates from a logit regression for a pooled sample, ${\hat{\beta}}^{\ast }$, the independent contribution of *k*_1_ To the rural-urban gap can be expressed as,

4 $$\frac{1}{N^U}{\sum}_{i=1}^UF\left({\hat{\alpha}}^{\ast }+{k}_1^R{\hat{\beta}}_1^{\ast }+{k}_2^R{\hat{\beta}}_2^{\ast}\right)-F\left({\hat{\alpha}}^{\ast }+{k}_1^U{\hat{\beta}}_1^{\ast }+{k}_2^R{\hat{\beta}}_2^{\ast}\right)$$

Similarly, the contribution of *k*_2_ Can be expressed as: -.

5 $$\frac{1}{\ {N}^U}{\sum}_{i=1}^UF\left({\hat{\alpha}}^{\ast }+{k}_1^U{\hat{\beta}}_1^{\ast }+{k}_2^R{\hat{\beta}}_2^{\ast}\right)-F\left({\hat{\alpha}}^{\ast }+{k}_1^U{\hat{\beta}}_1^{\ast }+{k}_2^U{\hat{\beta}}_2^{\ast}\right)$$

The contribution of each variable to the gap is thus equal to the change in the average predicted probability from replacing urban distribution with rural distribution while holding the distributions of the order variable constant.

However, the assumption of equal sample size is rarely true in practical situations. Since the rural sample is substantially larger, a large number of random subsamples of rural under-five mortality (equal size to total urban sample) are drawn to match each of them to the urban sample and calculate separate decomposition. Finally, the mean value of all these separate decomposition estimates is used as an approximate decomposition for the entire rural sample. Decomposition results are based on 100 replications.

All analyses of this study were carried out using STATA 16 [22].

## Results

### Trend of under-five mortality in India by place of residence, 1992–2021

**Fig. 1:**
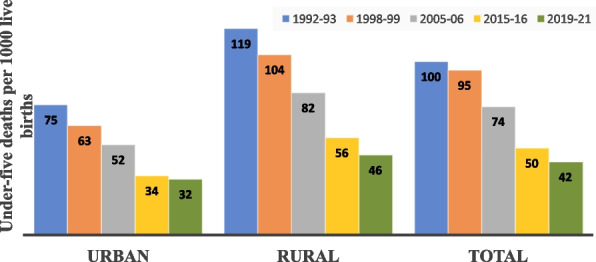
Under-five Mortality rate in India by place of residence (5 years prior to the survey); 1992–2021

It is observed that under-five mortality decreased slowly from 1992 to 93 to 2005–06 (100 to 74 per thousand live births), and after 2005–06 to 2015–16, it decreased faster (74 to 50) (Fig. 1). During 2005–06 to 2015–16, rural and urban under-five mortality decreased faster than any other period. However, from 2015 to 16 to 2019–21, it has decreased slowly (50 to 42). A similar trend was found in both urban and rural under-five mortality. Rural under-five deaths decreased from 119 to 46, and urban under-five deaths fell from 75 to 32 per thousand live births. Urban under-five mortality reduced from 75 to 52 from 1992 to 2006, 52 to 34 from 2006 to 2016, and 34 to 32 from 2016 to 2021. From 1992 to 93 to 2019–21, the annual decrease in rural under-five mortality is 1.6%, whereas, in case of rural under-five mortality, it is 2.7%. The rural-urban gap in under-five mortality has reduced from 44 per thousand in 1992–1993 to 30 per thousand in 2004–2005 which further decreased to 14 per thousand in 2019–2021. Despite reducing rural under-five deaths more rapidly than urban under-five deaths, it is still higher than urban area.

### State level variation in rural-urban gap in under-five mortality

**Fig. 2:**
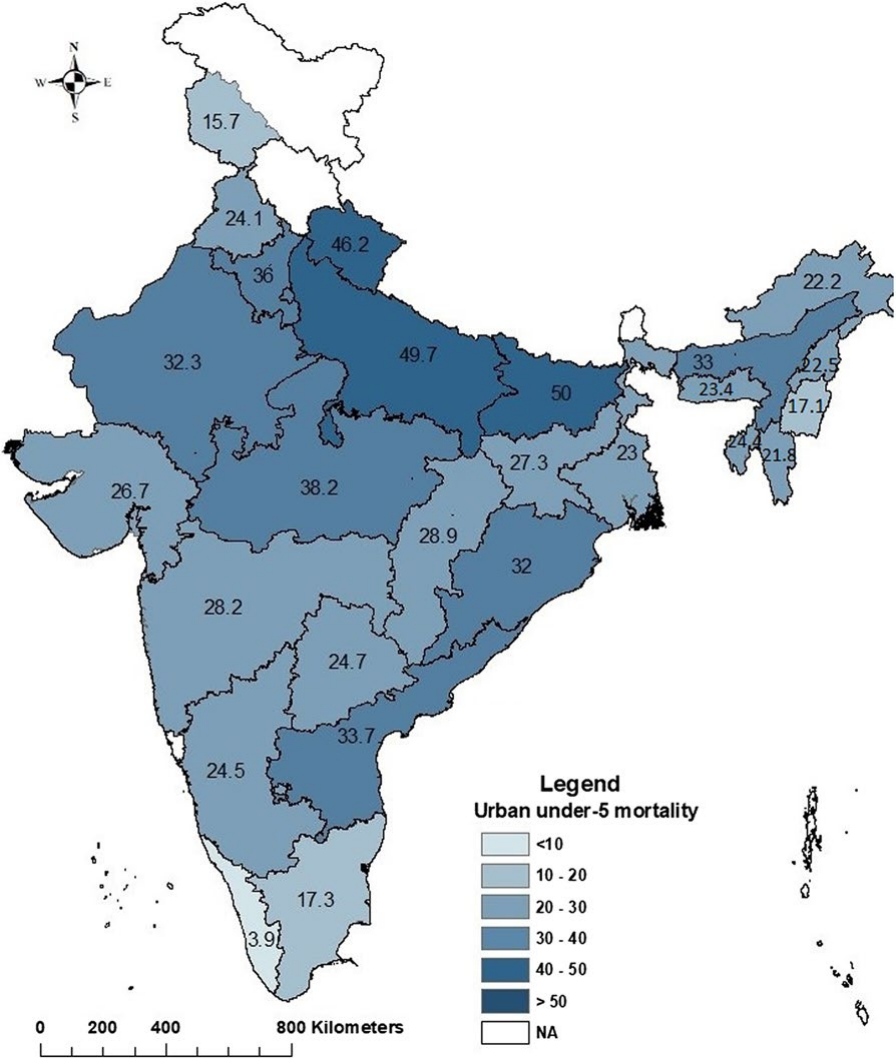
Under-five mortality in urban India, 2019–21

**Fig. 3:**
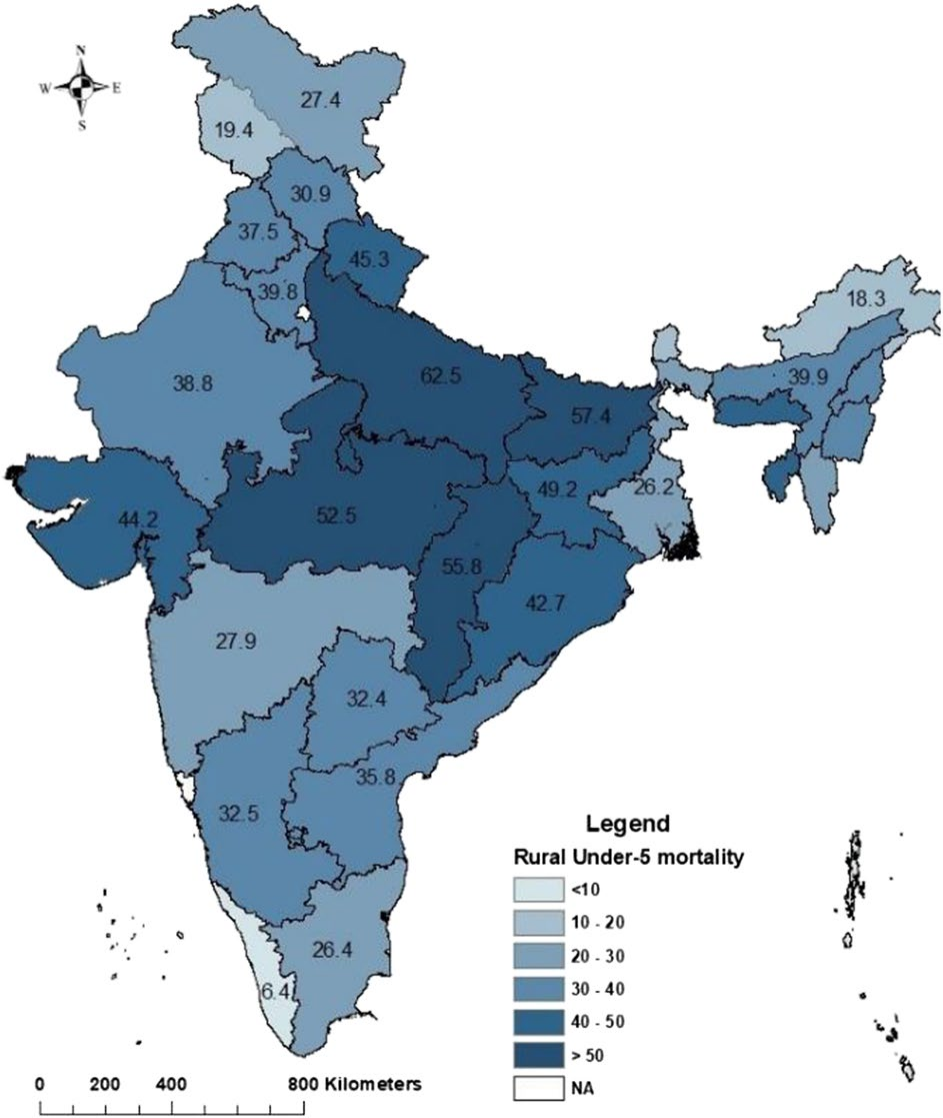
Under-five mortality in rural India, 2019–21

Figure 2 and Fig. 3 shows the state-wise under-five mortality by place of residence in India. In urban areas [Fig. 2], under-five mortality is the highest in Bihar (50), followed by Uttar Pradesh (49), Uttarakhand (46) and under-five mortality is lowest in Kerala (4), followed by Manipur (17), Tamil Nadu (17). There is no state where urban under-five mortality is more than 50 per thousand live births. Bihar, Uttar Pradesh, and Uttarakhand have more than 46 urban under-five deaths per 1000 live births. In the south, except Andhra Pradesh and northeast India except Assam, urban under-five mortality is comparatively lower. However, urban mortality is extremely low in the extreme south and northern states. A total of fourteen states (Jammu and Kashmir, Punjab, Arunachal Pradesh, Manipur, Tripura, Mizoram, Meghalaya, Nagaland, West Bengal, Puducherry, Telangana, Karnataka, Tamil Nadu, Kerala) achieved SDG recommendation of under-five mortality rate (25 per 1000 live births) in the urban area. That means almost 48% states have achieved the recommended SDG goal in under-five mortality in urban area.

While in rural areas [Fig. 3], under-five mortality is highest in Uttar Pradesh (62), followed by Bihar (57), Chhattisgarh (56), Madhya Pradesh (52) and under-five mortality is lowest in Kerala (6), followed by Sikkim (18), Arunachal Pradesh (19). Rural under-five mortality is in an undesirable position in the north-central states of India. EAG states are in significantly lagged conditions. Only five (Jammu and Kashmir, Sikkim, Arunachal Pradesh, Kerala, Andaman & Nicobar Islands) states achieved SDG recommendation of under-five mortality in the rural area. That means only 16% states have achieved the recommended SDG goal in under-five mortality in rural area.

The rural-urban gap in under-five mortality is maximum in Chhattisgarh (26.9), followed by Tripura (24.6), Jharkhand (21.9), Meghalaya (19.2), Manipur (19.1), Gujarat (17.5), Nagaland (14.3). Lowest gaps are found in Andhra Pradesh (2.1) followed by Kerala (2.5), West Bengal (3.2), Jammu & Kashmir (3.7), Haryana (3.8), Mizoram (4.4). Interestingly there are some union territories and states where rural mortality is lower than urban mortality. Those union territories and states are Dadra & Nagar Haveli and Daman & Diu (− 13.4) followed by Arunachal Pradesh (− 3.9), Uttarakhand (− 0.9) and Maharashtra (− 0.3) [Additional file 1: Table A1].

### The gross and net effect of place of residence on under-five mortality

Table 1 shows the sample characteristics of under-five deaths in India by demographic and socioeconomic variables. Female children die at a lower rate than male children, with approximately 4.20% dying compared to 4.62% for male children. Under-five mortality is higher when mothers give birth before 20 years, which is 4.92% compared to those whose mother’s age is 20 to 29 or after 29 years, which is 3.84 and 3.49% respectively. Second birth orders or birth intervals greater than 24 months have lower under-five mortality than first birth orders, second birth orders, third or more birth orders, or birth intervals less than 24 months. In terms of socioeconomic variables, under-five mortality is higher in rural areas than in urban areas; about 4.62% of rural under-five mortality compared with 3.28% of urban under-five mortality. Under-five mortality in higher educated mothers is 2.23% compared to whose mothers belong to primary and secondary are 4.93 and 3.52% respectively. Likewise, children whose mothers belong to a higher wealth index is low under-five mortality than poorest. Under-five mortality in the central (5.78%) and eastern parts of India (5.59%) is higher compared to the west (3.44%) and southern India (3.17%).

**Table 1:** Sample characteristics of under-five deaths in India by demographic and socioeconomic variables, (2019–21)

Variables	Sample Size(N)	Percent of under-five death
**Demographic variables**		
**Sex of Child**		
Male	2,69,535	4.62
Female	2,46,422	4.2
**Birth Interval**		
First Birth	1,84,472	4.22
2nd order birth & BI< 24 months	47,088	5.67
2nd order birth & BI> = 24 months	1,10,601	2.77
3rd or more order birth and BI< 24 months	53,674	8.73
3rd or more order birth and BI> = 24 months	1,20,122	3.84
**Mother’s age at first birth**		
Less than 20 years	2,82,613	4.92
20–29 years	2,20,492	3.84
More than29 years	12,852	3.49
**Socio-economic Variables**		
**Place of Residence**		
Urban	1,08,727	3.28
Rural	4,07,230	4.72
**Maternal Education**		
No Education	1,84,441	5.65
Primary Education	83,944	4.93
Secondary Education	2,12,042	3.52
Higher Education	35,530	2.23
**Mass Media Exposure**		
No Exposed	1,64,476	5.62
Partially exposed	3,46,198	3.89
Exposed	5283	1.76
**Religion**		
Hindu	3,79,214	4.74
Muslim	72,679	3.68
Christian	41,251	3.34
Other	22,813	3.42
**Caste**		
SC	1,02,090	5.27
ST	1,05,626	4.35
OBC	1,95,533	4.52
Other	1,12,708	3.54
**Wealth Index**		
Poorest	1,46,738	5.9
Poorer	1,23,025	4.74
Middle	99,493	3.99
Richer	81,939	3.29
Richest	64,762	2.54
**Other**		
**Region**		
East India	96,119	5.59
South India	64,947	3.17
North East India	78,328	3.32
Central India	1,31,187	5.78
North India	98,449	3.64
West India	46,927	3.44
**Total**	**5,15,957**	**4.19**

Table 2 shows the factors associated with under-five mortality in India. Model 1 shows the place of residence is a significantly associated factor in under-five mortality. The odds of dying under age 5 is 0.55 times more among rural children compared to urban children [OR:1.55, CI: 1.46,1.64]. After introducing other demographic and socio-cultural variables in model 2, the odds of death remained higher among rural children compared to urban children [OR:1.15, CI: 1.08,1.22]. However, in model 3, after controlling the wealth index, resident becomes an insignificant factor in under-five mortality. It indicates that wealth index of the household is more important factor than the place of residence.

**Table 2:** Results from Logistic regression model showing factors associated with under-five mortality in India (2019–21)

	Odd Ratio (95% confidence interval)		
Under-five Mortality	Model 1	Model 2	Model 3
**Place of Residence**			
Urban @R			
Rural	1.55***[1.46,1.64]	1.15***[1.08,1.22]	1.03 [0.97,1.10]
**Demographic variables**			
**Sex of Child**			
Male @R			
Female		0.90***[0.87,0.93]	0.90***[0.87,0.93]
**Birth Interval**			
First Birth @R			
2nd order birth & BI< 24 months		1.28***[1.20,1.36]	1.28***[1.20,1.35]
2nd order birth & BI> = 24 months		0.61***[0.58,0.64]	0.61***[0.58,0.65]
3rd or more order birth and BI< 24 months		1.71***[1.63,1.81]	1.71***[1.62,1.80]
3rd or more order birth and BI> = 24 months		0.72***[0.68,0.76]	0.72***[0.68,0.76]
**Mother’s age at first birth**			
Less than 20 years @R			
20–29 years		0.89***[0.85,0.93]	0.90***[0.86,0.94]
> 29 years		0.96 [0.84,1.10]	0.98 [0.85,1.12]
**Socio-economic variables**			
**Maternal Education**			
No Education @R			
Primary Education		0.93**[0.88,0.98]	0.94*[0.89,1.00]
Secondary Education		0.72***[0.68,0.75]	0.76***[0.73,0.81]
Higher Education		0.46***[0.41,0.51]	0.54***[0.48,0.60]
**Mass Media Exposure**			
No Exposed @R			
Partially exposed		0.90***[0.86,0.94]	0.95*[0.90,0.99]
Exposed		0.64**[0.46,0.88]	0.70*[0.51,0.97]
**Religion**			
Hindu @R			
Muslim		0.81***[0.76,0.86]	0.82***[0.77,0.87]
Christian		0.86*[0.75,1.00]	0.89 [0.77,1.02]
Other		0.95 [0.84,1.07]	1 [0.89,1.13]
**Caste**			
SC @R			
ST		0.96 [0.90,1.03]	0.94 [0.88,1.01]
OBC		0.93**[0.88,0.97]	0.95*[0.91,1.00]
Other		0.87***[0.82,0.93]	0.91**[0.85,0.97]
**Wealth Index**			
Poorest @R			
Poorer			0.93**[0.88,0.98]
Middle			0.86***[0.81,0.92]
Richer			0.74***[0.69,0.81]
Richest			0.64***[0.58,0.70]
**Other**			
**Region**			
East India @R			
South India		0.75***[0.70,0.81]	0.80***[0.74,0.86]
North East India		0.86***[0.79,0.94]	0.84***[0.77,0.92]
Central India		1.12***[1.07,1.18]	1.16***[1.10,1.22]
North India		0.83***[0.78,0.88]	0.92**[0.86,0.98]
West India		0.71***[0.66,0.78]	0.75***[0.69,0.82]

* *p <* 0.05, ** *p <* 0.01, *** *p <* 0.001

The female odds of under-five mortality are lower among children than male children [OR: 0.90 CI: 0.87,0.93]. The odds of dying under age 5 is more than first order birth when the birth interval is less than two years [For 2nd order OR:1.28, CI: 1.20,1.35; for 3rd or more OR: 1.71, CI: 1.62, 1.80]. Whereas this is less than first order birth when the birth interval is more than two years [For 2nd order OR:0.61, CI: 0.58,0.65; for 3rd or more OR: 0.72, CI: 0.68, 0.76].

Maternal education and mass media exposure are positively associated with under-five mortality. Now other backward classes [OR: 0.95, CI: 0.91, 1.00] and other forward caste [OR: 0.91, CI: 0.85, 0.97] are less likely to die groups under age five than the scheduled caste. After controlling other variables, Muslim children have significantly lower odds of under-five mortality compared to Hindu children [OR:0.82, CI: 0.77, 0.87]. Children born to mothers with the richest wealth quintile were less likely to die before age five than those born to the poorer mothers [OR: 0.64, CI: 0.58,0.70].

Compared to the Eastern part of India, other regions (except Central India) have lower likelihood of under five deaths.

Table 3 presents the detailed decomposition of the rural-urban gap in under-five mortality by the exposure variables. For simplicity, we have multiplied the coefficient by 100. More than 80% of the average rural-urban gap was explained by the variables considered in the analysis. Household wealth, maternal education, and exposure to media, were the main contributors to the difference in rural-urban gap under-five mortality in NFHS-5. For instance, in the case of the average rural-urban difference in under-five mortality, the contribution of household wealth is 50.82% and that of maternal education is 28.57%. The exposure to media is 11.48%. The region of residence also contributes 8.37% to the total rural urban gap. The wealth index is the most significant contributor to the rural-urban gap in under-five mortality than other factors in NFHS-5.

**Table 3:** Decomposition of the rural-urban gap in under-five mortality, National Family Health Survey 2019–21

Variables	Coefficient	%
**Demographic Variables**		
Sex of Child	−0.00007	− 0.52
Birth Interval	−0.00115	−8.16
Mother’s age at first birth	0.00051	3.58
**Socio-economic Variables**		
Maternal education	0.00404	28.57
Mass Media Exposure	0.00163	11.48
Religion	0.00035	2.47
Caste	0.00048	3.39
Wealth Index	0.00719	50.82
**Other**		
Region	0.00118	8.37
**Total**	**0.0142**	**100.00**
**Difference (Rural-Urban)**	**0.0171**	
**Percent Explained**	**82.80**	
**Percent Unexplained**	**17.20**	

## Discussion

The present study investigated the changing role of place of residence in under-five mortality. Earlier studies found that under-five mortality gap between rural and urban in India is due to rural-urban disparity in socioeconomic and demographic variables [23]. Our study finds that urban children still have survival advantage from NFHS 4 (1992–93) to NFHS 5 (2019–21) at national level and in majority of the states. Yet, the gap is reducing over time. Some earlier studies documented that reduction in the rural-urban gap in under-five mortality from 1992 to 93 to 2019–21 is due mainly to the improved household wealth, maternal education, mass media exposure, transport connectivity, health infrastructure in rural India [15, 23, 24].

However, regression analysis shows that rural urban status is a significant factor until when we do not control the role of the wealth index of the household. This indicates that there is no disadvantage for the rural children due to their place of residence if they belong to economically well-off household or their mothers are educated. This is contrast to some previous studies which found that rural children experience higher level mortality even after controlling other socioeconomic and demographic variables [8]. The disappearance of rural disadvantage may be due to the introduction of National Rural Health Mission and the improved health facilities in India in rural areas.

Decomposition analysis demonstrates that wealth index and educational attainment of the mothers are the most two important factors which contribute 75% of the total gap in under-five mortality by place of residence. Both are associated with better child care practices. The economic condition of the children’s household is linked to better nutrition and access to health care facilities [10]. Wealth differential is the most contributing factor for rural-urban under-five mortality gaps. Under-five mortality is lower among mothers belonging to a rich economic background and vice versa. As rural households are relatively poorer than urban households, household wealth index contributes more to under-five mortality. There was a substantial increase of the middle wealth quintile onwards in urban India and people with the lowest and second-lowest wealth quintile increased in rural India [25]. .

Unequal attainment of maternal education also increased the rural-urban gap in under-five mortality [26, 27]. According to the 2011 census, rural literacy is 68.91%, and the urban literacy rate is 84.98% [28]. According to the NFHS report, the mother’s literacy rate in India increased from 1992 to 2021. The female literacy rate in India is increasing from 43% to 72% from 1992 to 2021. Rural literacy is increasing from 34% to 72.7%, and the urban literacy rate is increasing from 67% to 87.3% [29]. In rural India, median number of female years of schooling completed is 4 years and in urban areas, it is 7.5 years [29]. A higher level of education of women in community level and higher attainment of mother’s own education seemed to play an important role in decreasing under-five mortality [30]. An educated mother would ensure routine health check-ups, timely vaccination, proper hygiene, and a nutritious diet for her children, resulting in low morbidity and mortality [31].

Unequal maternal exposure to mass media has also increased the rural-urban gap in under-five mortality. From the media exposure, a mother can know about the different sanitation practices, usefulness of breastfeeding, proper dietary habits for infants and other healthcare utilization programs [32–35]. In rural areas, women are less exposed to mass media. In rural India, 49.9% of women are not regularly exposed to any media, whereas, in urban, it is only 23.2 [29]. Earlier studies also showed that maternal exposure to mass media has a positive effect on reducing child mortality [14, 36].

### Limitations

Our study has one limitation. While our focus is to examine rural-urban gap in under-five mortality, we did not investigate the intra-urban mortality variation. Within urban area, there is wide difference in mortality indicators between slum and non-slum dwellers.

## Conclusion

Though India has witnessed a considerable decline in rural under-five mortality rate from 1992 to 93 and 2019–21, yet the total number of under-five children’s deaths is huge. The rural population still contributes higher proportion of the deaths. The existing rural-urban gap in under-five mortality suggests that the social and health policies should focus more on the rural areas. Our finding shows that economic and education differential is the main reason for under-five mortality in rural area. This call for attention to ensure that the future programme must lay on emphasis on mother from economically and educationally disadvantageous section. While there should be more emphasis on equal access to health care facilities by the rural population, there should be also effort to strengthen rural economy and quality of education. There is need to strengthen health care services in rural areas by improving the availability of trained human resource, physical infrastructure, medicines, and medical equipment..

## Supplementary Information

**Additional file 1.**

## Data Availability

The datasets generated and/or analysed during the current study are available in the Demographic Health Survey repository, [https://dhsprogram.com/data/available-datasets.cfm.]

## Abbreviations

OR: Odds ratio
CI: Confidence Interval
