# Supplementary material for: An update on explaining the rural-urban gap in under-five mortality in India
Source: BMC Public Health. 2022 Nov 16;22:2093. doi: 10.1186/s12889-022-14436-7 (PMC9670513; doi:10.1186/s12889-022-14436-7)
Supplement: Supplementary file 1 — Additional file 1. [file 12889_2022_14436_MOESM1_ESM.pdf]

Appendix

Table A1: State wise rural-urban gap in under-five mortality in India, NFHS-5 (2019-21)

STATE	Total	Urban	Rural	Rural-Urban Gap
Andaman & Nicobar Island	24.5	NA	9.5	9.5
Andhra Pradesh	35.2	33.7	35.8	2.1
Arunachal Pradesh	18.8	22.2	18.3	-3.9
Assam	39.1	33.0	39.9	6.9
Bihar	56.4	50	57.4	7.4
Chandigarh	NA	NA	NA	NA
Chhattisgarh	50.4	28.9	55.8	26.9
Dadra & Nagar Haveli and Daman &Diu	37.0	43.8	30.4	-13.4
Delhi	30.6	30.5	NA	NA
Goa	10.6	NA	NA	NA
Gujarat	37.6	26.7	44.2	17.5
Haryana	38.7	36.0	39.8	3.8
Himachal Pradesh	28.9	NA	30.9	NA
Jammu & Kashmir	18.5	15.7	19.4	3.7
Jharkhand	45.4	27.3	49.2	21.9
Karnataka	29.5	24.5	32.5	8.0
Kerala	5.2	3.9	6.4	2.5
Ladakh	29.5	NA	27.4	NA
Lakshadweep	NA	NA	NA	NA
Madhya Pradesh	49.2	38.2	52.5	14.3
Maharashtra	28.0	28.2	27.9	-0.3
Manipur	30.0	17.1	36.2	19.1
Meghalaya	40.0	23.4	42.6	19.2
Mizoram	24.0	21.8	26.2	4.4
Nagaland	33.0	22.5	36.8	14.3
Odisha	41.1	32.0	42.7	10.7
Puducherry	3.9	4.1	NA	NA
Punjab	32.7	24.1	37.5	13.4
Rajasthan	37.6	32.3	38.8	6.5
Sikkim	11.2	NA	17.8	NA
Tamil Nadu	22.3	17.3	26.4	9.1
Telangana	29.4	24.7	32.4	7.7
Tripura	43.3	24.4	49.0	24.6
Uttar Pradesh	59.8	49.7	62.5	12.8
Uttarakhand	45.6	46.2	45.3	-0.9
West Bengal	25.4	23.0	26.2	3.2
	41.9	32.0	46.0	

NA=Not applicable due to smaller sample size [fewer than 25 unweighted cases]
